# Clinical reasoning in intensive care: Insights from novice and expert physiotherapists

**DOI:** 10.4102/sajp.v82i2.2300

**Published:** 2026-04-30

**Authors:** Hudaa Kariem, Liezel Ennion, Farhana Karachi, Shamila Gamiet, Danelle Hess

**Affiliations:** 1 Department of Physiotherapy, Faculty of Community and Health Science, University of the Western Cape, Cape Town, South Africa; 2 Interprofessional Education Unit, Faculty of Community and Health Science, University of the Western Cape, Cape Town, South Africa

**Keywords:** clinical reasoning, expert, intensive care, novice, qualified clinicians, undergraduate physiotherapy students

## Abstract

**Background:**

Clinical reasoning in the intensive care unit (ICU), a complex, high-acuity and stressful environment, may require a different set of critical thinking and decision-making approaches to undergraduate physiotherapy student training in this setting that needs to be explored.

**Objectives:**

To explore clinical reasoning processes of undergraduate physiotherapy students (novices) and clinicians (experts) in ICU settings in the Western Cape.

**Method:**

A qualitative exploratory study using semi-structured interviews with purposively sampled participants: seven final-year physiotherapy novices and four physiotherapy clinicians with over 5 years of ICU experience. Data were analysed using Braun and Clarke’s six-phase thematic content analysis.

**Results:**

Both novice and expert physiotherapists described using information gathering, assessment planning, and hypothesis formulation from medical folder reviews and objective assessments to guide critical thinking and decision-making in ICU care. Experienced clinicians relied on the International Classification of Function framework and extensive ICU exposure to strengthen their reasoning. Novice physiotherapists reported that theory and its application supported their clinical reasoning, but their effectiveness was limited by the complex, high-pressure ICU environment, emotional burden of critically ill and end-of-life care, and feelings of overwhelm, fear, stress, anxiety and limited experience.

**Conclusion:**

The study highlights shared foundations but clear differences in the depth of clinical reasoning between novice and expert physiotherapists, with clinicians’ greater experience and situational familiarity enabling more advanced reasoning in ICU care.

**Clinical implications:**

Structured ICU exposure, emotional support and guided reasoning frameworks are needed to help novice physiotherapists apply theory effectively in high-pressure ICU settings.

## Introduction

Clinical reasoning (CR) is the cognitive process by which physiotherapists collect, analyse and synthesise patient information to make informed decisions (Hage et al. 2022). While clinical reasoning refers to how clinicians think (cognitive and metacognitive processes), clinical decision-making is what clinicians decide to do following the underlying interpretive process of reasoning (Hage et al. 2022). In physiotherapy, effective CR is essential for safe, high-quality care, particularly in intensive care units (ICU), where rapid decisions may influence survival and recovery (Gosselink et al. 2008). However, there is limited evidence on how CR is developed and enacted by undergraduate physiotherapy novices in ICU settings globally and in South Africa.

The ICU is a complex, high-acuity and stressful environment in which physiotherapists manage critically ill patients with multiple comorbidities, advanced monitoring and life-support technologies (Van Aswegen, Patman & Plani 2017). The physiotherapists’ role has long been reported as critical in the ICU multidisciplinary team and includes respiratory management, early mobilisation and prevention of ICU-acquired weakness, all of which require ongoing risk–benefit evaluation (Hanekom et al. 2011; Norrenberg & Vincent 2000). Recent work continues to highlight the central role of physiotherapists in early mobilisation, respiratory care, effective patient communication, and goal setting and functional recovery, reinforcing the need for robust CR skills in this setting (Amundadottir et al. 2017; Karachi et al. 2023). Clinical decisions must integrate pathophysiology, functional status, haemodynamic stability and ventilatory support within a multidisciplinary team. Therefore, these differences in the physiotherapeutic management of critically ill patients may require a different set of skills to develop clinical reasoning in physiotherapy novices and clinicians working in the ICU.

Within physiotherapy, CR typically involves problem identification, hypothesis generation and testing, and treatment planning across assessment and intervention (Jones, Jensen & Edwards 2008). Both novice and expert physiotherapists use hypothetico-deductive reasoning, but experts rely on rapid pattern recognition supported by experiential knowledge (Doody & McAteer 2002; Jensen et al. 2000).

Over time, changes in knowledge organisation allow experts to recognise familiar clinical patterns and act efficiently, whereas novices depend more on explicit, rule-based reasoning (Christensen et al. 2017; Rivett & Higgs 1997). For undergraduate physiotherapy novices, this involves transferring classroom learning into a setting characterised by uncertainty, high stakes and unfamiliar equipment. Clinical reasoning development in such environments depends not only on knowledge, but also on opportunities for guided practice, supervision, feedback and reflection (Schön 1983; Wijbenga et al. 2020).

Most work on CR in physiotherapy has focused on musculoskeletal and neurological practice, with less attention to cardiorespiratory and critical care settings (Smith, Higgs & Ellis 2007b; Smith, Higgs & Ellis 2008). The distinctive features of ICU practice, including technological complexity and the emotional burden of caring for critically ill patients, suggest that CR processes may differ from those in other areas of physiotherapy. Understanding how physiotherapy novices and experienced physiotherapy clinicians describe and perform CR in the intensive care clinical environment can inform curriculum design and clinical education strategies that better prepare novices for critical care practice.

Gaining insight into clinical reasoning processes from both novice and expert perspectives, and identifying potential gaps between these viewpoints, could inform improvements in physiotherapy clinical education for intensive care practice. Therefore, this paper describes the exploration of undergraduate physiotherapy students (novices) and clinicians (experts) perceptions of their clinical reasoning processes and practices in intensive care settings in the Western Cape, South Africa.

## Research methods and design

### Research design

A qualitative approach and exploratory descriptive design were used and conducted during the coronavirus disease 2019 (COVID-19) pandemic (2021–2022).

### Research setting

The research setting included a physiotherapy department at a university in the Western Cape from which the student population was sampled. Two central hospitals with ICUs in the Western Cape, which are the only sites where undergraduate physiotherapy novices from the participating university receive ICU clinical training, were approached to participate in the study. Only one central hospital agreed to participate, from which the physiotherapy clinicians working in these ICUs were recruited. The central hospital has a total of five Adult ICUs with nine physiotherapy clinicians who work across these ICUs. The clinical education at the university at which the study was conducted allows for novices to obtain 2 weeks of intensive ICU theory and practical knowledge and skills, after which novices are provided with a 5-week or 6-week ICU clinical block rotation where they are able to integrate theoretical and practical knowledge and skills into ICU practice and patient management.

### Population and sampling

Nine physiotherapy clinicians employed at the central hospital working across the five ICUs formed the population (*N* = 9) of this study. The student population included 61 final-year undergraduate physiotherapy novices registered in the physiotherapy department at the University of the Western Cape in 2022. A purposive non-probability sampling method was used with the following eligibility criteria. Physiotherapy clinicians were eligible if they worked in one or more of the ICUs and had ≥ 5 years of ICU working experience. Final-year novices who had completed an ICU rotation at either hospital during this period (February 2022 – June 2022) were eligible to participate in the study. Those who had not completed an ICU rotation were excluded. A sample of 11 participants was considered sufficient to generate rich, in-depth data consistent with qualitative thematic analysis recommendations (Vasileiou et al. 2018).

### Instrumentation

Data were collected through semi-structured interview guides self-developed by the primary researcher for physiotherapy clinicians and physiotherapy novices, respectively (Online Appendix 1). The clinician guide was informed by literature on expert–novice CR and focused on how clinicians understood and enacted CR in ICU, including information gathering, assessment, hypothesis generation and treatment planning (Case, Harrison & Roskell 2000; Edwards et al. 2004; Wainright et al. 2011). The student guide was informed by literature related to decision-making and clinical reasoning in physiotherapy and explored novices’ understanding of CR in ICU, their experience of applying theoretical knowledge in practice, and perceived facilitators and barriers to CR during ICU (Hess 2021; Langridge, Roberts & Pope 2016; Major et al. 2020; Smith et al. 2007a; Widerström, Rasmussen-Barr & Boström 2019; Wijbenga 2018). Both guides used open-ended questions and allowed participants to elaborate freely, ensuring coverage of key areas.

### Data collection

Four physiotherapy clinicians working in the ICU (*n* = 4) and 36 final-year undergraduate physiotherapy novices who had rotated through the ICUs at the time of data collection were eligible to participate in the study. The four physiotherapy clinicians and 36 undergraduate novices were all invited to participate in the study via email through the head of the physiotherapy department at the university, and the included central hospital, requesting them to contact the primary researcher if interested in participating in the study. Individual face-to-face audio-recorded interviews with physiotherapy clinicians were conducted by the primary researcher between April 2022 and June 2022. Individual interviews were conducted with novices online via Zoom by the researcher between July 2022 and October 2022, after completion of their ICU rotations. All interviews were conducted in English by the primary researcher, lasting approximately 45 min – 60 min. To minimise hierarchical influence in student interviews, the researcher emphasised her non-evaluative role, clarified that participation would not affect assessment, and used neutral, open-ended prompts.

### Data analysis

All audio-recorded interviews were transcribed verbatim using Otter.ai. All audio recordings were listened to by the primary researcher in order to check the accuracy of the transcriptions that were then manually analysed using the reflexive six-step thematic analysis by Braun and Clarke (2006, 2012). The primary researcher read all the transcripts a few times to familiarise herself with and immerse herself in the data. A hybrid deductive–inductive approach was used. Deductive codes were informed by existing CR literature (e.g. information gathering, hypothesis generation, pattern recognition, safety and/or contraindications, learning strategies), whilst inductive codes captured unanticipated but key ideas (e.g. emotional burden, impact of online learning). Whilst initial coding was informed by established clinical reasoning constructs (e.g. information gathering, hypothesis generation), several salient patterns emerged inductively from the data, particularly relating to emotional burden, ICU overwhelm and the impact of pandemic-related curricular disruption.

This iterative process allowed for the discovery of unanticipated but significant themes and insights. Furthermore, authors distinguished CR from clinical decision-making within the analysis by coding cognitive and metacognitive processes related to how the participants think as clinical reasoning. The authors coded what participants decided to do following the underlying interpretive process of reasoning as clinical decision-making during the analysis process. During analysis, data were differentiated between clinical reasoning and clinical decision-making. Clinical reasoning was analysed as a process-oriented activity, reflected in participants’ accounts of interpreting patient information (e.g. use of records and charts, identification of risks and contraindications, judgements of physiological stability, and problem prioritisation). These data captured how clinical understanding was constructed and justified. Clinical decision-making was analytically identified at points where participants articulated a commitment to action, including the selection, modification, deferral, or withholding of physiotherapy interventions. This distinction allowed the analysis to account for instances in which extensive clinical reasoning occurred without immediate intervention. Transcripts were coded manually by the primary researcher in Microsoft Word, focusing on the explicit content of participants’ accounts. Clinician and student transcripts were initially coded separately to allow group-specific patterns to emerge. The codes from both clinician and student transcripts were then collated into potential subthemes and themes; similar codes were merged, and redundant codes were removed. Themes were reviewed and refined in relation to coded data and the dataset. The coded transcripts were reviewed by two of the other authors (Danelle Hess and Shamila Gamiet) familiar with the topic and with experience on qualitative research serving a form of debriefing that offered a different perspective on the data and encouraged reflexivity. In the final phase, themes were named, defined and reported on with illustrative quotations to demonstrate how participants described their CR processes and the factors that facilitated or hindered these processes in ICU.

### Trustworthiness

The insider position was acknowledged, and a reflexive journal was maintained to document assumptions, emotional responses and potential sources of bias and improve the credibility of the findings (Hsiung 2008). During student interviews, the researcher adopted a non-evaluative stance, reassured novices that participation was voluntary and confidential, and used neutral prompts to reduce hierarchical influence. All participants were invited to provide input on the transcribed data and preliminary analysis, confirming that the transcriptions captured the essence of what participants had said during the interviews (member checking) (Anney 2014, Graneheim & Lundman 2004, Lincoln & Guba 1985). Two clinicians and two novices responded and confirmed that the data that was transcribed and analysed reflected what they had said.

### Ethical considerations

Ethics approval was obtained from the University of the Western Cape’s (UWC) Biomedical Research and Ethics Committee (BMREC) (BM 21/9/20). Additional permissions were secured from the UWC Registrar, Head of Physiotherapy Department, hospital departments at Groote Schuur and Tygerberg hospitals, the Department of Health, and the National Health Research Database.

All participants were provided with an information sheet outlining the nature and purpose of the study. Written informed consent was provided by all participants sent to the primary researcher via email. Participation was voluntary, with participants able to withdraw at any time without explanation. Novices were reassured that interviews were non-evaluative and designed solely to understand their clinical reasoning perspectives. Explicit informed consent was obtained from all participants for the audio-recording and secure transcription of interviews using Otter.ai.

Confidentiality was maintained through numeric coding of participants, secure data storage for 5 years, and exclusion of personal identifiers from recordings. The study respected all participants’ rights to dignity, confidentiality and privacy.

## Results

### Demographic details

A total of four (*N* = 4) clinicians and seven (*N* = 7) novices consented to participate in the study. Of the novices, three (*n* = 3) were males, and four (*n* = 4) were female. All four clinicians were female. The clinicians had a mean of 17 (standard deviation [s.d.] ± 5.9) years of experience as a qualified physiotherapist, mean 12.5 (s.d. ± 1.3) years of experience as a physiotherapy clinician in ICU and mean 12 (s.d. ± 4.7) years of experience with student teaching and all four (100%) had postgraduate training specifically in critical care (Table 1).

**TABLE 1:** Summary of demographic characteristics of study participants.

Participant number	Designation	Gender	Years of experience
C1	Clinician	Female	17
C2	Clinician	Female	17
C3	Clinician	Female	11
C4	Clinician	Female	14
S1	Student	Male	N/A
S2	Student	Female	N/A
S3	Student	Female	N/A
S4	Student	Male	N/A
S5	Student	Male	N/A
S6	Student	Female	N/A
S7	Student	Female	N/A

C, clinicians; N/A, not applicable; S, students.

### Qualitative findings

The analysis of qualitative data generated three main themes. These included: (1) Information gathering, assessment planning and hypothesis formulation in intensive care (deductive); (2) Enhancing critical thinking and decision-making in clinical reasoning in intensive care (deductive); (3) Constraints in critical thinking and decision-making in clinical reasoning in intensive care (deductive and inductive analysis).

#### Theme 1: Information gathering, assessment planning and hypothesis formulation in intensive care

This theme describes how the novices and clinicians gather information, plan and execute their assessment, formulate the problem list and hypothesis and make informed decisions regarding ICU patient management. The theme is divided into two subthemes.

**Subtheme 1.1: Patient medical record and bed chart assessment and problem-solving in intensive care:** Physiotherapy novices and clinicians described using patient records, clinical charts and observations to guide their assessments, identify precautions and contraindications, determine stability for intervention and prioritise problems, illustrating a structured, evidence-informed approach to clinical reasoning in the ICU (Table 2, quotes 1–4).

**TABLE 2:** Theme 1, subthemes, codes and verbatim quotes.

Themes	Subthemes	Codes	Verbatim quotes
**Theme 1:** Information gathering, assessment planning and hypothesis formulation in intensive care	**Subtheme 1.1:** Patient medical record and bed chart assessment and problem-solving in intensive care	patient folder, charts, doctor’s file, nursing notes medical history vital signs (trends) precautions -contraindications objective observation formulate problem list treatment goals and activities	C1: ‘Looking at the folder and getting an idea of what you can expect from the patient. Your focus of your assessment would be specific to that condition.’ C2: ‘… deciding are there any precautions … any contraindications … is the patient actually stable for intervention because it is an ICU setting.’ S2: ‘I would look in the patient’s folder, see why he is here … look at the chart … trends … then local observations… and then just see what you need to work on …’ S4: ‘So, I’d start with my doctor’s file, then look at the nursing notes, then I tend to my charts, then I tend to my patient. … then I’d start with my objective observation, then I’d start my chest assessment. Then I’d assess all that, and then I would formulate my problem list, so what was going to be my priority for that day, and what the problem was and then I would start treating according to that and also look at what I would like to do long term.’ C3: ‘I think we kind of lean towards the ICF or aspects of it … but I feel like I do rely on my own experiences …’ C1: ‘I’d say not formally, but subconsciously I’m using the ICF. I would say what the problem is, what the underlying reason … and then from that what can we do about it.’ C1: ‘As I’ve become more experienced, there is a lot I take in automatically … you immediately start to observe what’s going on … what is this thing that’s making my senses tingle?’ C2: ‘… areas you’ve worked in will allow you to judge situations … your own experience will guide you …’ C1: ‘I’ve done the advanced respiratory course … ICU courses and quite a bit in the way of teaching …’ C4: ‘I feel like it came with practice so the more time I spent in ICU I built up the knowledge base, so you use what you’ve done before, practice and experience helped’
	**Subtheme 1.2:** Applying theoretical frameworks and leveraging theoretical knowledge and clinical experience for clinical reasoning in intensive care	ICF and the aspect of ICF ‘subconscious’ framework usage -experiential pattern recognition tacit knowledge years of ICU exposure repeated case exposure -postgraduate training ongoing CPD teaching students	

C, clinician; CPD, Continuous Professional Development; ICF, International Classification of Function Framework; ICU, intensive care unit; S, student.

**Subtheme 1.2: Applying theoretical frameworks and leveraging theoretical knowledge and clinical experience for clinical reasoning in intensive care:** Physiotherapy clinicians highlighted that whilst they draw on theoretical frameworks such as the International Classification of Function (ICF), much of their clinical reasoning in the ICU is guided by accumulated experience, prior training and accumulative knowledge from courses attended, and repeated practice, which help them clinical reasoning to recognise problems instinctively (formulate problem list) and then make informed clinical decisions and judgements (treatment goals and activities) during ICU patient management (Table 2, quotes 5–10).

Theme 1 reflects deductively derived clinical reasoning processes that align with established hypothetico-deductive models of physiotherapy reasoning. Both novice and expert physiotherapists described systematic information gathering, assessment planning, and hypothesis formulation as foundational to ICU patient management. However, clinicians’ accounts demonstrated greater efficiency and selectivity in information processing, suggesting more advanced pattern recognition and prioritisation supported by experiential knowledge. In contrast, novices’ reasoning appeared more deliberate and sequential, reflecting reliance on explicit rules and theoretical knowledge. These differences are consistent with novice–expert theory and highlight how experience enables clinicians to manage the high informational demands of ICU environments more fluidly, reducing cognitive load during assessment and planning.

#### Theme 2: Enhancing critical thinking and decision-making in clinical reasoning in intensive care

This theme captures how novices develop the ability to think critically and make informed decisions (clinical reasoning ability) when managing ICU patients through the integration of theoretical knowledge, clinical exposure and guided support. It reflects their growing competence in interpreting complex clinical information, integrating theoretical knowledge with real-time observations, and justifying their clinical actions in a high-pressure environment. Novices described how prior learning provided a cognitive framework that helped them interpret complex patient presentations, anticipate potential risks and justify assessment and treatment choices.

These reasoning processes were foundational to subsequent decision-making in the ICU context, where actions were perceived as high-risk and closely linked to patient safety.

The theme also highlights the role of independent learning and guided practice and reflection through clinician support, strengthening novices’ clinical reasoning ability and confidence in ICU patient management and structured evidence-based decision-making. The theme is divided into two subthemes (Table 3).

**TABLE 3:** Theme 2, subthemes, codes and verbatim quotes.

Themes	Subthemes	Codes	Verbatim quotes
**Theme 2:** Enhancing critical thinking and decision-making in clinical reasoning in intensive care	**Subtheme 2.1:** Integrating theoretical knowledge and clinical exposure to enhance clinical reasoning in the ICU	application of knowledge using lecture content to understand ICU patient presentations ICU exposure real-life patient cases	S5: ‘I took the knowledge and actually applied it to the ICU …’ S7: ‘… the theory helps you to better understand and better reason … because you understand what you’re coming in to …’ S2: ‘What I find useful, definitely exposure … seeing the case in person just helps.’ S7: ‘The theory helps you to better understand and better reason, gives you better clinical reasoning, because you understand what you’re coming in to and what you should, what you will definitely be seeing.’ S4: ‘In the beginning … I was quite overwhelmed … so I prepared very well theoretically … whenever I didn’t know something I would immediately read up …’ S2: ‘… before block, I’ll try to go through content … that’s helped me a lot.’ S4: ‘My clinician and supervisor [*clinical educator*] … were always willing to help me learn, they would help me see the bigger picture and prompt me to develop my clinical reasoning.’ S2: ‘Being around other health professionals just helps because they can give you tips and tricks of things to remember the condition and that can help with your clinical reasoning.’
	**Subtheme 2.2:** Strategies for independent learning and utilisation of clinician support	prepared theoretically reading up on unfamiliar conditions revising content preparing before the block guidance from clinicians and clinical educators, other healthcare professionals	

ICU, intensive care unit; S, student.

**Subtheme 2.1: Integrating theoretical knowledge and clinical exposure to enhance clinical reasoning in the intensive care unit:** This subtheme captures how novices apply both prior learning and hands-on exposure to patient care in the ICU setting. Novices emphasised that applying theoretical knowledge through ICU patient presentations, particularly related to respiratory and cardiorespiratory physiotherapy, in the ICU environment was essential for developing their understanding and reasoning. They noted that theory enabled them to understand underlying pathophysiology, anticipate clinical problems, and organise assessment findings and provided a foundation that helped them anticipate and interpret clinical situations, whilst real-world exposure and seeing patients in person (seeing real cases) made the learning more meaningful and solidified their clinical reasoning and improved confidence. Clinical exposure thus reinforced this knowledge, allowing students to contextualise theoretical concepts through real patient encounters. Seeing patients in the ICU environment consolidated, reinforced and deepened their learning, helping them integrate theory with practice more effectively and making clinical reasoning more meaningful and improving confidence in interpreting clinical information (Table 3, quotes 11–14).

**Subtheme 2.2: Strategies for independent learning and utilisation of clinician support:** This subtheme emphasises both the novices’ independent learning strategies and how they engage with available clinical support to facilitate their clinical reasoning ability in the ICU. Novices reported feeling overwhelmed at the start of their ICU placements, which prompted active engagement in independent learning strategies and motivated them to prepare thoroughly before clinical blocks by revisiting theoretical content and reading up on unfamiliar topics. These independent learning strategies were described as supporting their reasoning processes by reducing uncertainty and increasing preparedness when encountering complex ICU patients. The proactive preparation helped them feel more equipped and supported their learning during the ICU clinical education block.

Novices emphasised the value of guidance and support from clinicians and clinical educators, noting that this support helped with reasoning development by helping novices interpret clinical information more holistically and refine their thinking. It helped them understand the broader clinical context and strengthened the development of their critical thinking, reasoning and decision-making skills and abilities in the ICU. Clinician prompts and feedback assisted novices in linking theoretical knowledge to patient-specific contexts, strengthening their clinical reasoning and, in turn, improving confidence in decision-making (Table 3, quotes 15–18).

Theme 2 highlights that clinical reasoning development in ICU novices is scaffolded through the integration of theoretical knowledge, repeated clinical exposure, and guided supervision. Novices relied on structured, theory-based reasoning characteristic of early-stage practice, with clinician support enabling progression towards more context-sensitive interpretation of patient information. Although decision-making actions were described, these consistently followed deliberate reasoning processes, indicating that decision confidence in the ICU was grounded in strengthened clinical reasoning rather than isolated technical skill acquisition.

#### Theme 3: Constraints in critical thinking and decision-making in clinical reasoning in intensive care unit patient management

Theme 3 captures how both the ICU environment and personal emotional responses constrained novices’ critical thinking, reasoning and decision-making. Novices described how stressful, high-pressure ICU setting characterised by constant alarms, complex technology and uncertainty around critically ill patients, made it difficult to think clearly and feel adequately prepared. These environmental pressures were compounded by personal factors such as fear, anxiety, self-doubt, emotional discomfort, and feeling overwhelmed or underprepared, particularly when managing end-of-life scenarios. Together, these constraints limited their ability to confidently and effectively engage in clinical reasoning in the ICU. The theme is divided into three subthemes (Table 4).

**TABLE 4:** Theme 3, subthemes, codes and verbatim quotes.

Themes	Subthemes	Codes	Verbatim quotes
**Theme 3:** Constraints in critical thinking and decision-making in clinical reasoning in ICU patient management	**Subtheme 3.1:** Environmental constraints influencing clinical reasoning in the ICU	stressful environment; alarms and monitors complex technology (ventilators) limited clinical preparedness for some conditions uncertainty of critically ill patients; -impact of COVID-19 and online learning	S4: ‘The stressful environment made it a bit difficult … when you are busy assessing a patient, and the alarms go off, your attention quickly turns.’ S6: ‘… the challenge I faced in ICU was the ventilators, we never saw one, I had to learn how it works.’ S4: ‘We started studying when COVID started … case-based learning helped, but it doesn’t fully prepare you for the environment.’ S4: ‘… I found it quite difficult to transfer at the start, because we started studying when COVID started, so I find the classroom and the clinical environment quite different. So, I usually transfer through case-based learning, that’s something they started early with us because of having to write online, but it doesn’t fully prepare you for the environment.’ S6: ‘… So that has been an adjustment, especially with us not having in-person class, so the techniques that we learned in first year had to be refreshed in clinical practice, especially this year.’ S2: ‘… everything’s just been online, so it’s much harder to understand things and go through things … It’s been difficult to transfer from the classroom to clinical practice.’ S4: ‘In the beginning … I was quite overwhelmed …’ ‘The fear of critically ill patients … going close to them, touching them, was uncomfortable.’ ‘… the fact of you treating a patient but they could just pass away …’ S1: ‘When I go to ICU … I feel like I underperformed the whole day …’ S4: ‘I think the stressful part is the monitors going off, and you know the severity of the condition, you are aware the whole time that you have to be careful with them …’ S5: ‘I think the only thing that got to me was me being anxious, the beeping machines, and also knowing that the patient’s life in some instances was a matter of life or death … because it was the first time being in an ICU setting.’ S7: ‘I was a bit nervous going into it. So that changed the how I, in the beginning, how I think would reason because of nerves.’
	**Subtheme 3.2:** Curricula constraints influencing clinical reasoning in the ICU	impact of COVID-19 and online learning The classroom and the clinical environment are quite different case-based learning not having an in-person class	
	**Subtheme 3.3:** Personal constraints influencing clinical reasoning in the ICU	overwhelmed fear uncomfortable anxiety stress feeling underperformance self-doubt emotional burden related to critically ill and end-of-life care	

COVID-19, coronavirus disease 2019; ICU, intensive care unit; S, student.

**Subtheme 3.1: Environmental constraints influencing clinical reasoning in the intensive care unit:** Novices highlighted that the ICU environment itself posed significant challenges to their learning and reasoning.

The constant noise and alarms created a stressful atmosphere that disrupted focus during patient assessment and observation. They also noted that unfamiliar equipment, particularly ventilators, added to the difficulty, as they had limited prior exposure to these technologies (Table 4, quotes 19–21).

**Subtheme 3.2: Curricular constraints influencing clinical reasoning in the intensive care unit:** Novices described difficulties transferring theoretical knowledge to the ICU because of predominantly online learning during COVID-19. Limited in-person instruction and practical exposure meant that previously learned techniques and concepts required refreshing, constraining their ability to apply knowledge confidently and effectively in the complex and intense real ICU clinical environment (Table 4, quotes 22–24).

**Subtheme 3.3: Personal constraints influencing clinical reasoning in the intensive care unit:** Novices reported that anxiety, fear, and stress in the ICU triggered by critically ill patients, life-or-death situations, and constant monitoring alarms constrained their clinical reasoning. Feelings of being overwhelmed, nervous, or underperforming affected their confidence and ability to think clearly, limiting their application of theoretical knowledge in real-time patient care (Table 4, quotes 25–31).

The findings from theme 3 suggest that emotional responses in ICU settings are not peripheral to clinical reasoning but are integral to how information is processed and prioritised. Fear, anxiety and perceived underperformance appeared to narrow attentional focus and increase cognitive load, potentially limiting students’ capacity to integrate multiple information sources simultaneously. This supports emerging views that affective factors are central components of clinical reasoning in high-stakes environments, particularly for novices.

Themes 1 and 2 aligned closely with established clinical reasoning constructs and were deductively analysed. Theme 3 emerged both deductively and inductively from participants’ accounts and captured unanticipated emotional, environmental and curricular constraints influencing novices’ clinical reasoning and decision-making in ICU contexts that are not explicitly foregrounded in traditional clinical reasoning models. Novices’ descriptions of fear, anxiety, overwhelm and perceived underperformance suggest that affective responses significantly shaped how clinical information was attended to, interpreted, and prioritised. These emotional responses appeared to increase cognitive load, narrowing attentional focus and constraining novices’ capacity to integrate multiple information sources simultaneously. In the ICU context, constant monitoring alarms, ventilator dependency, rapid physiological instability and proximity to end-of-life care intensified these affective demands, directly influencing both reasoning processes and subsequent decision-making. This finding extends existing clinical reasoning frameworks by highlighting the central role of emotional and contextual factors in shaping novice reasoning in high-acuity environments.

## Discussion

This study contributes to the growing qualitative evidence on how physiotherapy students (novices) and clinicians (experts) develop clinical reasoning within the complex and dynamic environment of intensive care. It underscores the need for a revised approach to both theoretical and clinical physiotherapy education. The study recognises how positive and negative environmental, educational/curricula and personal factors, as well as clinical exposure and experience shape novices’ and clinicians’ reasoning processes, and strengthen their capacity to apply effective critical thinking, reasoning and decision-making strategies in ICU practice. In this study, *clinical reasoning* is understood as the cognitive and interpretive processes through which physiotherapists gather, integrate and evaluate information, whereas *clinical decision-making* refers to the observable choices and actions that follow from these reasoning processes. Whilst closely interrelated in practice, this distinction guided the analysis, with themes reflecting influences on reasoning processes, decision outcomes, or both. A common starting point in the clinical reasoning process of both the novice and expert physiotherapist in ICU practice included information gathering, assessment planning and hypothesis formulation. Novices and experts used patient medical records and objective assessments to formulate hypotheses, identify problems and plan effective treatments for ICU patient management. This aligns with models of physiotherapy CR that emphasise systematic data collection as the foundation of decision-making. Both groups focused on diagnosis, medical history, trends in vital signs and precautions and contraindications as the process-oriented activities that captured how clinical understanding was constructed and justified to assist in deciding whether and how to manage the ICU patient (Edwards et al. 2004; Jones et al. 2008). This finding therefore suggests that ICU services should reinforce structured assessment protocols and ensure that physiotherapists, novice and expert, have consistent access to comprehensive patient records and monitoring data, supporting accurate hypothesis formulation and safe, evidence-based decision-making. With regard to physiotherapy, theoretical and clinical training in intensive care curricula, the findings suggest that academic and clinical educators should explicitly teach and scaffold systematic information gathering and hypothesis-driven assessment processes. This can be achieved by using case-based learning, simulation, virtual or augmented reality and real-life cases, as well as guided practice (clinician support) to strengthen novices’ ability to apply these core components of clinical reasoning in ICU settings.

The clinicians (expert) clinical reasoning processes could be deemed as somewhat superior to novice physiotherapists because of their years of clinical experience and exposure in the ICU and lifelong learning. Clinicians’ accounts reflected pattern recognition and experiential judgement, drawing on repeated exposure to similar cases and postgraduate training knowledge and information. They described ‘automatic’ observations at the bedside and intuitive responses to subtle changes in patient status, consistent with literature on expert clinicians’ integrated, non-analytical reasoning informed by tacit knowledge. Clinicians reported drawing sometimes explicitly and sometimes ‘subconsciously’ on the ICF, Disability and Health when identifying problems and planning interventions, framing reasoning around impairments, activity limitations and participation restrictions in ICU patient management.

Novices were not explicitly asked about their use of frameworks, and they did not spontaneously refer to the ICF when discussing clinical reasoning related to ICU patient management. It cannot, therefore, be concluded that they did not use the ICF; rather, the data suggest that they focused on course content and condition-specific knowledge when talking about their clinical reasoning development and process. This represents a limitation and indicates a need for research that explicitly explores how novices understand and apply frameworks such as the ICF, as structured frameworks can provide scaffolding for novice reasoning development (Christensen et al. 2017) in ICU practice. Nonetheless, the apparent gap between clinicians’ informal ICF-informed reasoning and novices’ emphasis on condition-specific theory suggests a potential educational opportunity. More explicit integration of ICF-based reasoning in ICU teaching and supervision may help bridge the transition from classroom frameworks to clinical application.

Novices identified several factors that enhanced their critical thinking, reasoning and decision-making in intensive care. They emphasised the role of prior theoretical knowledge, particularly respiratory and cardiorespiratory content, in helping them recognise problems and understand complex patient presentations.

Novices, by contrast, emphasised structured, theory-based thinking. They described applying classroom content to unfamiliar ICU presentations, revisiting lectures and notes, and using pathophysiological concepts to justify treatment choices. Their descriptions suggest a stepwise hypothetico-deductive process with strong reliance on formal theoretical knowledge and independent reading. The conclusion that novices used theory-based approaches is grounded in their self-reported preparation and decision-making rather than direct observation.

Independent learning strategies, such as revising notes, consulting guidelines and journals, and using online resources, were commonly used to address knowledge gaps before and during ICU blocks and were perceived to improve reasoning and confidence. Repeated exposure to the ICU environment and support from clinicians and clinical educators were also central to guiding their clinical reasoning ability and skills. Working with ICU patients daily helped novices move from generic diagnosis-focused thinking towards more patient-specific reasoning, whilst prompts and feedback from supervisors helped them to ‘see the bigger picture’ and refine their thinking.

These findings reinforce the importance of structured clinical education, mentorship, clinician support and feedback in scaffolding CR development in ICU practice and furthermore support the importance of fostering self-directed learning skills early in physiotherapy education to prepare novices for lifelong learning in clinical practice (Mann, Gordon & MacLeod 2017). The findings in theme 2 suggest that ICU clinical teams and educators should provide structured supervision, regular feedback, and repeated exposure to varied patient cases to help novices transition from theory-driven, stepwise reasoning to more nuanced, patient-specific clinical reasoning and decision-making, ultimately strengthening their confidence and performance in complex ICU care. Curricula should integrate strong foundational teaching in cardiorespiratory theory with opportunities for independent learning, case-based reasoning, and progressive clinical immersion, ensuring novices can effectively apply theoretical knowledge, address knowledge gaps and refine their clinical reasoning through guided practice and reflective support.

Novices reported environmental, curricula and personal constraints to their clinical reasoning development process. Thus, their ability to critically think and make decisions in the ICU, where rapid responses are required to sudden changes in critically ill patients’ conditions, is affected. The ICU environment was perceived as stressful and highly technological, with constant alarms and complex equipment affecting their ability to think clearly (Major et al. 2020).

Unfamiliarity with ventilators, monitoring devices and certain conditions contributed to a sense of under-preparedness. The legacy of COVID-19 and online learning was evident, with novices noting that reduced in-person teaching and limited early hands-on ICU practice made it harder to transfer classroom learning into ICU clinical practice. These concerns echo reports by Chesterton, Richardson and Tears (2022) of disrupted training affecting the development of practical clinical skills and highlight the particular vulnerability of cohorts whose formative learning occurred largely online. Novices described fear, anxiety and self-doubt, particularly when treating critically ill or end-of-life patients as personal constraints to their clinical reasoning development and process in the ICU.

Some reported leaving the ICU feeling that they had ‘underperformed’, even when they had completed assessments and treatments. These emotional responses appeared to interfere with their ability to reason clearly and confidently during ICU patient management. The prominence of emotional burden in novices’ accounts adds an important dimension to existing models of CR, which are often primarily cognitive. In environments such as the ICU, emotional reactions may be central influences on how information is noticed, interpreted and acted upon.

Addressing these affective dimensions may therefore be as important as teaching reasoning strategies and frameworks. These findings suggest that ICU teams should provide structured orientation, gradual familiarisation with technology, and supportive supervision that actively addresses novices’ emotional responses, helping reduce cognitive overload and enabling clearer reasoning and safer decision-making in high-pressure scenarios in the ICU. Physiotherapy programmes should strengthen hands-on ICU preparation through simulation, early skills training, and emotionally informed teaching approaches, incorporating debriefing, reflective practice, and resilience-building. This may then ensure novices can transfer theoretical knowledge into practice whilst managing the environmental, curricular, and emotional demands of ICU care.

Overall, physiotherapy clinical reasoning in the ICU is shaped by a complex interplay of theoretical knowledge, clinical experience, environmental pressures and emotional factors, underscoring the need for integrated educational and clinical strategies that strengthen foundational reasoning skills, support experiential learning, and address the affective challenges of critical care practice. These inductively derived findings extend existing clinical reasoning models by foregrounding emotional and environmental influences that are often implicit or underexplored, particularly in high-acuity settings such as the ICU.

### Strengths and limitations

The study is novel and provides evidence for physiotherapy clinical reasoning skills development required in a complex, dynamic and stressful clinical environment, such as the ICU in the South African context. Furthermore, the study findings are strengthened because of the multiple strategies that were used to enhance trustworthiness and eliminate hierarchical bias (including supervisor oversight, audit trails, member checking and reflexive journaling). However, the findings must be interpreted with some caution as the study has a few limitations. It involved a small, context-specific sample of four ICU clinicians and seven final-year novices from two tertiary hospitals in the Western Cape; findings may not be transferable to other institutions or settings. Finally, novices were not explicitly asked about their use of specific CR frameworks (such as the ICF), which limits conclusions about framework use in this cohort and should be considered when interpreting the findings. The primary researcher’s clinical experience in ICU physiotherapy may have shaped interpretations of students’ accounts, particularly in recognising subtle expressions of anxiety, uncertainty and perceived underperformance. Reflexive journaling and co-author review were used to critically examine these interpretations and minimise assumptions about competence or readiness.

## Conclusion

This study shows that whilst physiotherapy novices and experts share foundational reasoning processes, experts’ experience enables more nuanced, adaptive decision-making in ICU care.

The depth of clinical reasoning between novice and expert physiotherapists differs, with clinicians’ greater experience and situational familiarity enabling more advanced reasoning in ICU care.

Physiotherapy clinical reasoning in the ICU is shaped by a dynamic interaction of knowledge, experience, environment, and emotion. Whilst novices rely heavily on theoretical foundations and stepwise reasoning, their development is challenged by the technological intensity, stress and rapid decision-making demands of the ICU.

Repeated exposure, supportive supervision and opportunities to integrate theory with practice gradually strengthen their confidence and reasoning sophistication. Importantly, emotional factors profoundly influence how novices interpret and act on clinical information. Together, these insights highlight the need for holistic educational and clinical supports that cultivate cognitive and affective dimensions of ICU reasoning. Strengthening structured ICU exposure, emotional support, and guided reasoning frameworks is essential to help novices translate theory into confident, context-sensitive reasoning under the pressures of critical care.
